# Application of an artificial intelligence-based system in the diagnosis of breast ultrasound images obtained using a smartphone

**DOI:** 10.1186/s12957-023-03286-1

**Published:** 2024-01-02

**Authors:** Ryutaro Mori, Mai Okawa, Yoshihisa Tokumaru, Yoshimi Niwa, Nobuhisa Matsuhashi, Manabu Futamura

**Affiliations:** 1 Department of Breast Surgery, Gifu University Hospital, 1-1 Yanagido, Gifu, 501-1194 Japan; 2 Department of Gastroenterological Surgery Pediatric Surgery, Gifu University, Graduate School of Medicine, 1-1 Yanagido, Gifu, 501-1194 Japan

**Keywords:** Breast ultrasound, AI, Object detection, Smartphone, Breast cancer, Breast tumors

## Abstract

**Background:**

Breast ultrasound (US) is useful for dense breasts, and the introduction of artificial intelligence (AI)-assisted diagnoses of breast US images should be considered. However, the implementation of AI-based technologies in clinical practice is problematic because of the costs of introducing such approaches to hospital information systems (HISs) and the security risk of connecting HIS to the Internet to access AI services. To solve these problems, we developed a system that applies AI to the analysis of breast US images captured using a smartphone.

**Methods:**

Training data were prepared using 115 images of benign lesions and 201 images of malignant lesions acquired at the Division of Breast Surgery, Gifu University Hospital. YOLOv3 (object detection models) was used to detect lesions on US images. A graphical user interface (GUI) was developed to predict an AI server. A smartphone application was also developed for capturing US images displayed on the HIS monitor with its camera and displaying the prediction results received from the AI server. The sensitivity and specificity of the prediction performed on the AI server and via the smartphone were calculated using 60 images spared from the training.

**Results:**

The established AI showed 100% sensitivity and 75% specificity for malignant lesions and took 0.2 s per prediction with the AI sever. Prediction using a smartphone required 2 s per prediction and showed 100% sensitivity and 97.5% specificity for malignant lesions.

**Conclusions:**

Good-quality predictions were obtained using the AI server. Moreover, the quality of the prediction via the smartphone was slightly better than that on the AI server, which can be safely and inexpensively introduced into HISs.

## Background

Screening for breast cancer should be performed using mammography, based on clinical evidence [1]. However, mammography results in pain in the pressed breast and radiation exposure [2]. Moreover, the detection of breast cancer is sometimes difficult if the mammary gland on mammography is dense (dense breast), particularly in Japanese women [3]. Therefore, breast ultrasound (US) is frequently used for breast cancer screening in Japan, although its use of breast US for breast cancer screening has not yet been established. Although mammography can be interpreted by skilled physicians after being performed by radiology technicians, the diagnosis of lesions on breast US images must be determined by sonographers. Therefore, the psychological burden on sonographers is very heavy, and the false-positive rate of cancer screening using breast US is very high [4].

Deep learning is an emerging artificial intelligence (AI) technology that has been utilized in daily life. Image recognition is one of the most suitable fields for deep learning and is used in applications such as facial recognition [5], optical character readers [6], and cruise control systems for cars [7]. Three types of tasks are performed using image recognition with deep learning: “classification,” “object detection,” and “image segmentation” image segmentation [8]. Classification involves assigning only a class label to an image, which is simpler than object detection or segmentation. Object detection involves identifying the locations of the objects within an image and drawing a bounding box around them. Image segmentation involves identifying the boundaries of objects within an image and assigning a label to each pixel within the boundary; this requires numerous calculations. Among these tasks, object detection, which can detect both the class of objects and their location on an image with a relatively low number of calculations, is the most balanced image recognition task.

AI is also used in some areas of healthcare, especially for the analysis of medical imaging [9], and the associated prices are apparently low in product catalogs. However, most implementations of AI are combined with expensive testing equipment or department information systems such as gastrointestinal endoscopy [10] or picture archiving and communication systems (PACSs) [11], which ultimately incurs large expenses. Moreover, some AI diagnosis services require Internet access from hospital networks, which exposes hospital information systems (HISs) to malware threats (e.g., computer viruses or ransomware) [12]. Therefore, safe implementation of AI diagnosis technologies at a low cost must be established.

**Fig. 1:**
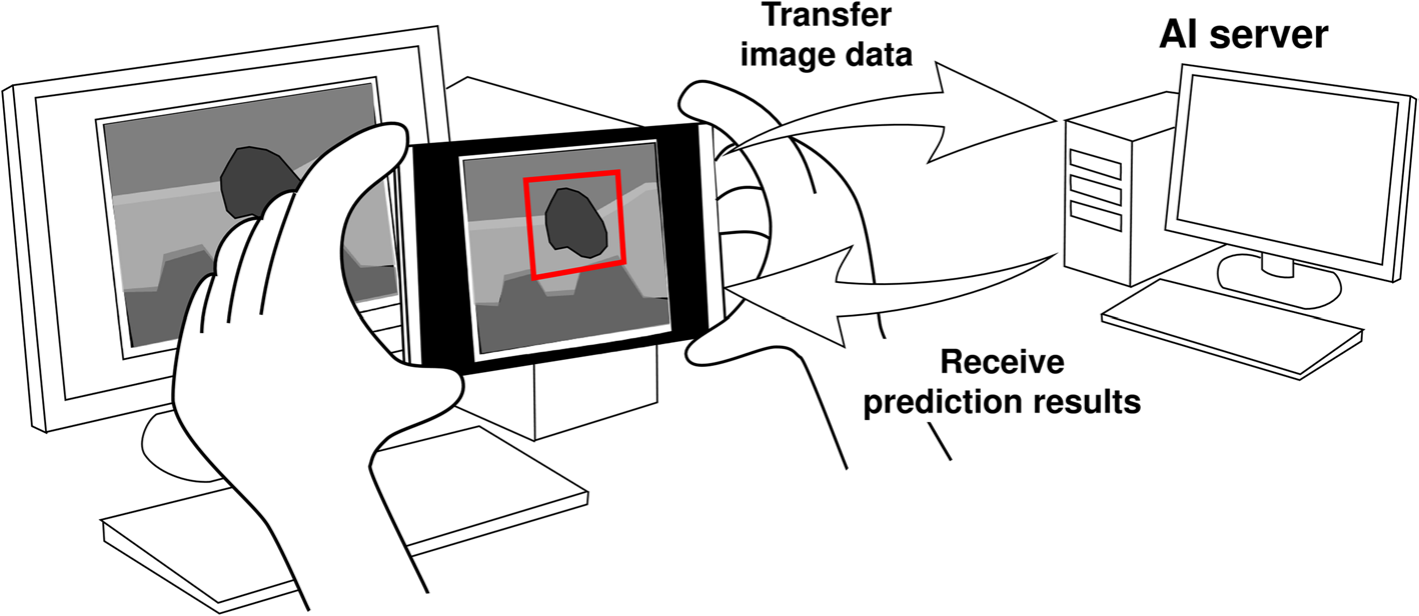
The inexpensive and safe way of introducing AI diagnosis techniques into clinical practice. The user captures the ultrasound image displayed on the monitor. Then, the results of the prediction performed on the AI server are drawn as rectangles on the screen of the smartphone

In this study, we investigated the feasibility of using AI to predict lesions on breast US images using object detection algorithms. Moreover, we explored an inexpensive and safe system that applies AI to the diagnosis of medical images captured using a smartphone camera in a clinical setting (Fig. 1).

## Patients and methods

### Patients and images

The records of patients with breast cancer who underwent breast US at Gifu University Hospital between 2017 and 2018 were reviewed, and breast US images showing breast cancer or benign tumors were collected. Images without lesions were collected for validation. The following US equipment were used in this study: GE Healthcare LOGIC E9 and HITACHI Avius.

### Annotation of lesions

**Fig. 2:**
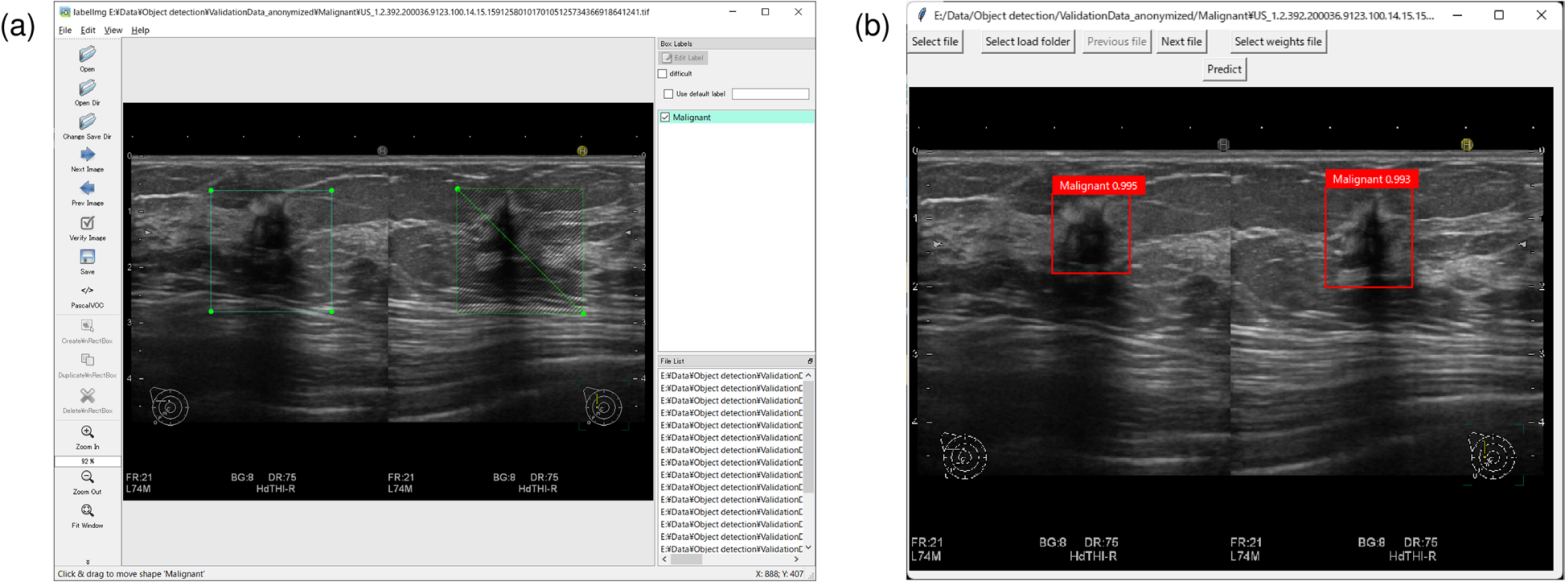
The annotation tool and graphical user interface (GUI) for object detection. **a** Annotation with LabelImg. **b** GUI for object detection

The lesions on the collected images were divided into two classes: “malignant” and “benign.” To obtain training data for the object detection algorithm, which consists of the classes of the lesions and the coordinates of rectangles surrounding the area of the lesions, we used the LabelImg software program, which is an open-source software (OSS) program published on GitHub [13] (Fig. 2a).

### Environment of AI server for performing deep learning and prediction

We used a computer with an Intel Xeon E3-1270v3 3.50 GHz central processing unit, a GeForce GTX 1060 graphics processing unit (GPU) with 6 GB of video random access memory, and a Windows 10 operating system. To use the Python programming language on the AI server, Anaconda version 1.10.0, which is a Python distribution, was used [14]. The following programs were used to construct the deep learning model: TensorFlow version 1.6.0, which is a library for deep learning with a deep neural network distributed as an OSS by Google [15], and Keras version 2.2.4, which is a wrapper for TensorFlow that allows the easy creation of deep learning models [16].

### Models of object detection

In this study, we used the OSS object detection models of You Only Look Once v3 (YOLOv3) [17] for the prediction of lesions in breast US images. Before machine learning, the pre-trained data built by the authors were applied to the YOLOv3 model.

### Evaluation of the accuracy of the prediction on AI server

To evaluate the accuracy of the prediction on the AI server, we developed a graphical user interface (GUI) that displayed the results of the prediction by drawing rectangles with red (malignant) or green (benign) strokes on the images (Fig. 2b). The accuracy of the prediction was evaluated using images spared from the training set for validation purposes. Sensitivity and specificity were then calculated.

The sensitivity of the malignant lesions was calculated by dividing the number of images predicted as malignant among the images of malignant lesions by the total number of images of malignant lesions. Specificity was calculated as the sum of the number of images predicted as benign lesions (or images with no lesions) divided by the total number of images of benign lesions and images with no lesions. The sensitivity and specificity for benign lesions were calculated in the same way as for malignant lesions.

### Development of a smartphone application for capturing US images and displaying the prediction results

A smartphone application was constructed using JavaScript as the web application. The “getUserMedia” method was used to capture US images displayed on the HIS monitor using a smartphone camera. The captured images were resized to 416 × 416 dots and converted into base64 strings. The images were then transferred to the application programming interface (API), as described later, using the WebSocket protocol. To display the prediction results on a smartphone screen, this application also receives the results of the prediction performed on the AI server from the API and draws rectangles according to the received results.

### Development of API for data transmission between a smartphone and the AI server

A smartphone application cannot connect directly to an AI server because of firewalls. Therefore, we developed an API that transmits data between a smartphone and AI server. A virtual server with an Ubuntu 20.04.3 LTS operating system was proposed on a public cloud. Then, Nginx version 1.18.0, which is a web server application, and Node.js version 10.19.0, which is the JavaScript runtime environment, were installed on the server. The application for data transmission on this server was built using Socket.io, version 2.3.0., which is the wrapper library for the WebSocket protocol.

### Development of application for data transmission between the API and the AI server

A Python application running on an AI server was built using Socket.io for Python version 0.5.7.4. to receive image data from the API and transfer the prediction results to the API.

### Evaluation of the predictive accuracy of the smartphone application

The quality of the prediction obtained using a smartphone to capture images was also investigated using the validation data described above. The image on the monitor was captured by a smartphone camera so that the image filled the screen of the smartphone, and the light in the room was turned off to avoid the reflection of light when capturing images of the monitor. The sensitivity and specificity were calculated in the same way as described above.

## Results

### The patients and collected images

**Fig. 3:**
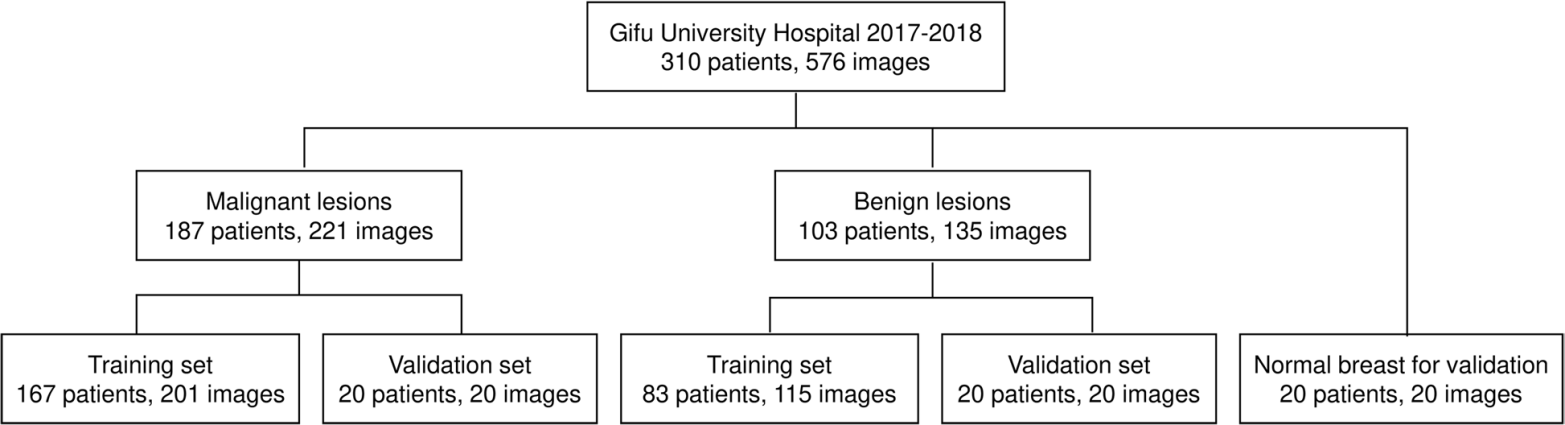
Cohort selection flowchart of training and validation datasets

We collected 221 images of malignant lesions from 187 patients and 135 images of benign lesions from 103 patients. From these images, 20 images of malignant lesions and 20 images of benign lesions were randomly selected and used for validation. These images were not included in the training data. Twenty images of normal breast tissues from 20 patients were collected for validation. The selections are shown in Fig. 3.

### Overview of the established AI and the accuracy of the prediction by the AI server

**Fig. 4:**
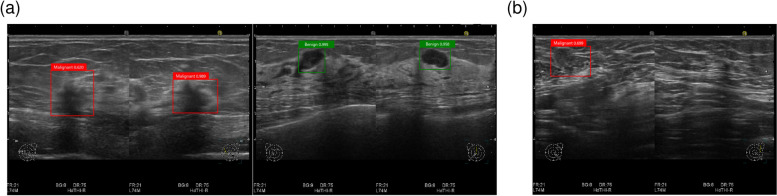
Examples of the detection on an AI server. **a** Successful examples. **b** A failed example

The time spent on machine learning by the YOLOv3 model using our training data was approximately 50 min. Typical lesions on the images could be detected, and malignant or benign lesions could be distinguished (Fig. 4a). However, some lesions could not be detected, or the background was mistakenly detected as lesions (Fig. 4b). The speed of the prediction on the AI server was 12 ps. The quality of the prediction on the AI server was investigated using the validation data. The confusion matrix, sensitivity, and specificity are presented in Tables 1 and 2. Sensitivity and specificity for malignant lesions were 100% and 75%, respectively.

**Table 1:** The confusion matrix of prediction on the AI server

	Predicted				
	Malignant	Benign	Both	None	Total
Actual					
Malignant	18	0	2	0	20
Benign	1	15	4	0	20
Normal	4	1	1	14	20

**Table 2:** The sensitivity and specificity of prediction on the AI server

	Sensitivity	Specificity
Malignant	100.00%	75.00%
Benign	95.00%	90.00%
Normal	100.00%	70.00%

### Prediction via smartphone and its accuracy

**Fig. 5:**
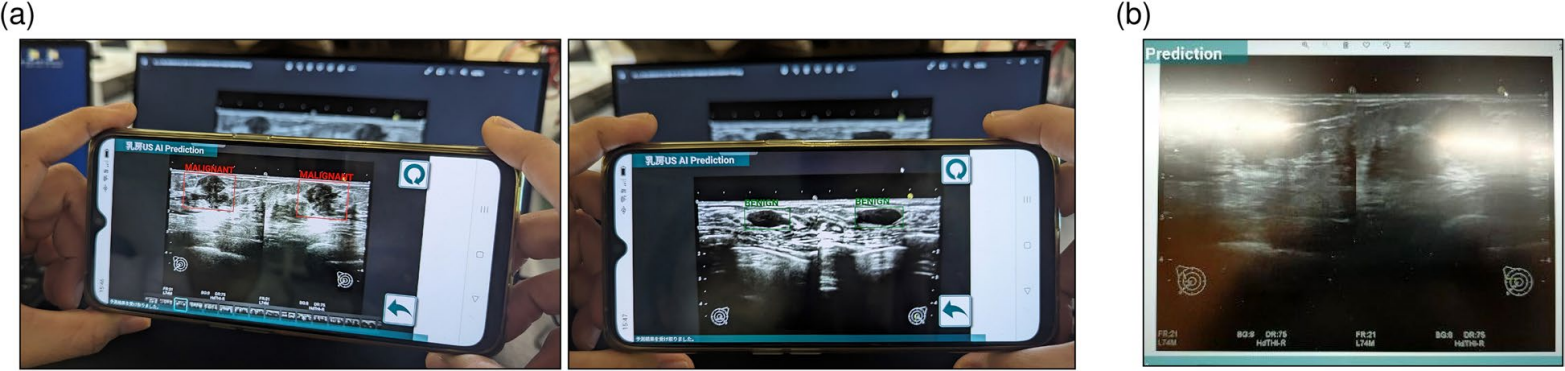
Examples of the detection via smartphone. **a** Successful examples. **b** A failed example

Typical lesions in the images could be detected, and malignant or benign lesions could be distinguished by prediction using images captured by a smartphone (Fig. 5a). Because the reflection of room light disturbed the detection of lesions (Fig. 5b), the room light needed to be turned off. The confusion matrix, sensitivity, and specificity of the results are presented in Tables 3 and 4. Sensitivity and specificity for malignant lesions were 100% and 97.5%, respectively.

**Table 3:** The confusion matrix via smartphone

	Predicted				
	Malignant	Benign	Both	None	Total
Actual					
Malignant	16	0	4	0	20
Benign	0	20	0	0	20
Normal	1	1	0	18	20

**Table 4:** The sensitivity and specificity of prediction via smartphone

	Sensitivity	Specificity
Malignant	100.0%	97.5%
Benign	100.0%	87.5%
Normal	100.0%	90.0%

## Discussion

In the present study, we developed a deep-learning-based AI prediction method for breast US using object detection algorithms with relatively small training data, which resulted in good sensitivity and specificity. Moreover, we developed an AI prediction method using a smartphone to capture US images displayed on a HIS monitor. The quality of the prediction using images obtained via the smartphone was slightly higher than that of the prediction using the AI server, although we were concerned about a decrease in the quality.

Deep-learning-based prediction of breast US has recently been studied. Most studies on the use of AI in breast ultrasound have focused on the differentiation of benign and malignant breast masses based on the B-mode ultrasound features of the masses [18]. Various models of object detection have been used in studies of breast ultrasound, such as fully convolutional one-stage object detection (FCOS), Faster Regions with Convolutional Neural Networks (R-CNN), single-shot multibox detector (SSD), YOLO, and YOLOv3 [19, 20] and the quality of the prediction in these studies was good, indicating that object detection was a suitable model for the AI-based diagnosis of breast US. The quality of the prediction in our study was similar to that of previous studies, although the indicators of quality were different from those of our study. Image segmentation has also been used to predict lesions in breast US images, and this model can theoretically predict the area of the lesions more concisely [21, 22]. However, this model requires a computer with a high-specification GPU because of the heavy computational burden. Therefore, the use of object detection models appears to be the most appropriate measure for the implementation of AI in the diagnosis of breast US.

The introduction of AI-based systems for the diagnosis of medical images in clinical practice is challenging. As described in the Introduction section, most research on AI applications has been conducted in the business setting, and some systems require the hospital network to be accessible to the Internet, which is associated with high implementation costs and risk of malware infection [23]. In the system described in the present study, only a typical smartphone and its internet access were required for implementation, and there was no need to connect to the hospital network. Accordingly, the inexpensive system that we describe is safer than the existing AI-based diagnosis systems. Therefore, we believe that the system we established, which uses images captured on a smartphone, could contribute to the utilization of AI-based diagnostic technologies.

The utilization of smartphones in AI-based diagnosis systems has been studied in dermatology, ophthalmology, respirology, and other fields [24]. In these studies, lesions on the body surface or visible areas of the body (rather than an HIS monitor) were captured by a smartphone camera, and the disease was diagnosed by an AI-powered recognition system on the smartphone or through an API. To the best of our knowledge, our study is the first to propose the use of an AI-based diagnosis system that uses medical images displayed on the monitor of an HIS captured by a smartphone camera.

The present study is associated with several limitations. Although we obtained relatively good results, the size of the training data was small and the accuracy of the prediction was insufficient, especially for benign lesions. Larger training datasets are required to improve prediction accuracy. Standardization of capturing conditions should be established for stable AI prediction using smartphones. Furthermore, the speed of prediction is insufficient, and a computer with a high-specification GPU is required if many physicians use the system.

## Conclusions

We propose an inexpensive and safe method for introducing AI-based diagnosis of medical images captured using a smartphone in clinical practice, without technical innovation. Our system will contribute to the development of similar AI-based systems in clinical practice.

## Data Availability

All the data would be supplied by the corresponding author if required.

## Abbreviations

AI: Artificial intelligence
API: Application programming interface
FCOS: Fully convolutional one-stage object detection
GUI: Graphical user interface
GPU: Graphics processing unit
HIS: Hospital information system
OSS: Open-source software
PACS: Picture archiving and communication system
R-CNN: Regions with convolutional neural networks
SSD: Single-shot multibox detector
US: Ultrasound
YOLO: You Only Look Once

## References

[1] Schopper D, de Wolf C (2009). How effective are breast cancer screening programmes by mammography? Review of the current evidence. Eur J Cancer.

[2] Miglioretti DL, Lange J, van den Broek JJ, Lee CI, van Ravesteyn NT, Ritley D, Kerlikowske K, Fenton JJ, Melnikow J, de Koning HJ, Hubbard RA (2016). Radiation-induced breast cancer incidence and mortality from digital mammography screening: a modeling study. Ann Intern Med.

[3] Boyd NF, Guo H, Martin LJ, Sun L, Stone J, Fishell E, Jong RA, Hislop G, Chiarelli A, Minkin S, Yaffe MJ (2007). Mammographic density and the risk and detection of breast cancer. N Engl J Med.

[4] Ohuchi N, Suzuki A, Sobue T, Kawai M, Yamamoto S, Zheng YF, Shiono YN, Saito H, Kuriyama S, Tohno E, Endo T, Fukao A, Tsuji I, Yamaguchi T, Ohashi Y, Fukuda M, Ishida T, J-START investigator groups (2016). Sensitivity and specificity of mammography and adjunctive ultrasonography to screen for breast cancer in the Japan Strategic Anti-cancer Randomized Trial (J-START): a randomised controlled trial. Lancet.

[5] Baltrušaitis T, Robinson P, Morency L. OpenFace: an open source facial behavior analysis toolkit. 2016 IEEE Winter Conference on Applications of Computer Vision (WACV). 2016. p. 1-10.

[6] Garg S, Gupta KK, Prabhakar N, Garg AR, Trivedi A (2018). Optical character recognition using artificial intelligence. Int J Comput Appl.

[7] Nie Z, Farzaneh H (2022). Real-time dynamic predictive cruise control for enhancing eco-driving of electric vehicles, considering traffic constraints and signal phase and timing (Spat) information, using artificial-neural-network-based energy consumption model. Energy.

[8] Khan A, Al-Habsi S (2020). Machine learning in computer vision. Proc Comput Sci.

[9] Bajwa J, Munir U, Nori A, Williams B (2021). Artificial intelligence in healthcare: transforming the practice of medicine. Future Healthc J.

[10] El Hajjar A, Rey JF (2020). Artificial intelligence in gastrointestinal endoscopy: general overview. Chin Med J (Engl).

[11] Leiner T, Bennink E, Mol CP, Kuijf HJ, Veldhuis WB (2021). Bringing AI to the clinic: blueprint for a vendor-neutral AI deployment infrastructure. Insights Imaging.

[12] Neprash HT, McGlave CC, Cross DA, Virnig BA, Puskarich MA, Huling JD, Rozenshtein AZ, Nikpay SS (2022). Trends in ransomware attacks on US hospitals, clinics, and other health care delivery organizations, 2016–2021. JAMA Health Forum.

[13] GitHub - HumanSignal/labelImg. https://github.com/tzutalin/labelImg. Accessed Sept 2019.

[14] Anaconda | The world’s most popular data science platform. https://www.anaconda.com/. Accessed Sept 2019.

[15] TensorFlow. https://www.tensorflow.org/. Accessed Sept 2019.

[16] GitHub - keras-team/keras: Deep learning for humans. https://github.com/keras-team/keras. Accessed Sept 2019.

[17] Redmon J, Divvala S, Girshick R, Farhadi A. You only look once: unified, real-time object detection. arXiv:150602640 [cs]. Published online May 9 2016.

[18] Wu GG, Zhou LQ, Xu JW, Wang JY, Wei Q, Deng YB, Cui XW, Dietrich CF (2019). Artificial intelligence in breast ultrasound. World J Radiol.

[19] Wang Y, Lin X, Zhang X, Ye Q, Zhou H, Zhang R, Ge S, Sun D, Yuan K (2022). Improved FCOS for detecting breast cancers. Curr Med Imaging.

[20] Cao Z, Duan L, Yang G, Yue T, Chen Q (2019). An experimental study on breast lesion detection and classification from ultrasound images using deep learning architectures. BMC Med Imaging.

[21] Yap MH, Pons G, Marti J, Ganau S, Sentis M, Zwiggelaar R, Davison AK, Marti R, Yap MH, Pons G, Marti J, Ganau S, Sentis M, Zwiggelaar R, Davison AK, Marti R (2018). Automated breast ultrasound lesions detection using convolutional neural networks. IEEE J Biomed Health Inform.

[22] Kumar V, Webb JM, Gregory A, Denis M, Meixner DD, Bayat M, Whaley DH, Fatemi M, Alizad A (2018). Automated and real-time segmentation of suspicious breast masses using convolutional neural network. PLoS One.

[23] Khan B, Fatima H, Qureshi A, Kumar S, Hanan A, Hussain J, Abdullah S. Drawbacks of artificial intelligence and their potential solutions in the healthcare sector. Biomed Mater Devices. 2023. p. 1–8. Published online February 8. 10.1007/s44174-023-00063-2 PMC9908503 36785697

[24] Susanto A. P. Winarto H. Fahira A. Abdurrohman H. Muharram A. P. Widitha U. R. Warman Efirianti G. E. Eduard George Y. A. Tjoa K. Building an artificial intelligence-powered medical image recognition smartphone application: what medical practitioners need to know. Inform Med Unlocked. 2022;32:101017.

